# Management of health information of nepalese labour migrants

**DOI:** 10.1186/s12992-023-00927-8

**Published:** 2023-04-25

**Authors:** Rajendra Karkee, Minani Gurung, Lisasha Poudel, Chiranjivi Baral, Pratik Adhikary, Radheshyam Krishna KC, Sundip Gurung, Vasil Gajdadziev, Patrick Duigan, Montira Inkochasan, Kolitha Prabhash Wickramage, Ganesh Gurung

**Affiliations:** 1 School of Public Health and Community Medicine, BP Koirala Institute of Health Sciences, Dharan, Nepal; 2 Nepal Institute of Development Studies (NIDS), Kathmandu, Nepal; 3 Institute for Social and Environmental Research-Nepal, Chitwan, Nepal; 4 International Organization for Migration (IOM), Kathmandu, Nepal; 5 IOM Regional Office for Asia and Pacific Region (ROAP), Bangkok, Thailand; 6 IOM, Geneva, Switzerland

**Keywords:** Labour, Migration, Management, Health, Nepal

## Abstract

**Introduction:**

The monitoring and improvement of the health of labour migrants (LMs) require sufficient health data to be recorded and managed. In this context, this study was conducted to explore the management of health information of Nepalese labour migrants (NLMs).

**Methods:**

This is an explorative qualitative study. Stakeholders involved directly or indirectly in maintaining the health profile of NLMs were first mapped, physically visited, and any documents or information were collected. Then, sixteen key informant interviews were conducted among these stakeholders related to labour migrants’ health information management and challenges. A checklist extracted information from the interviews, and a thematic analysis was carried out to summarize the challenges.

**Results:**

Government agencies, non-governmental organizations, and government approved private medical centers are involved in generating and maintaining the health data of NLMs. The Foreign Employment Board (FEB) records deaths and disabilities of NLMs while at work abroad and these health records are also maintained in an online portal called Foreign Employment Information Management System (FEIMS) under the Department of Foreign Employment (DoFE). Health assessment of NLMs is a mandatory procedure before departure, which is done through the government-approved pre-departure private medical assessment centers. The health records from these assessment centers are first recorded in paper-based form and then entered into an online electronic form to be stored by the DoFE. The filled-up paper forms are sent to District Health Offices, which further report the data to the Department of Health Services (DoHS), Ministry of Health and Population (MoHP) and associated governmental infectious diseases centers. However, there is no formal health assessment of NLMs upon arrival to Nepal. Key informants raised various issues and concerns in maintaining health records of NLMs, which were grouped into three themes: lack of interest to develop a unified online system; need of competent human resources and equipment; and developing a set of health indicators for migrant health assessment.

**Conclusion:**

The FEB and government-approved private assessment centers are the main stakeholders in keeping the health records of outgoing NLMs. The current migrant health record keeping procedure in Nepal is fragmented. The national Health Information Management Systems does not effectively capture and categorize the health record of NLMs. There is a need to effectively link national health information system with premigration health assessment centers; and potentially develop a migrant health information management system by systematically keeping health records electronically with relevant health indicators on departing and arriving NLMs.

## Introduction

Migration is an inevitable phenomenon in the 21st century. About 3.5% of the world’s population (272 million) is international migrants, of which 164 million are migrant workers in 2019 [1]. One of the reasons for migration is employment, and the migrants for this purpose are termed as labour migrants (LMs) or migrant workers [2]. LMs can be vulnerable to various health risks because they are usually casual, unskilled, uneducated, or low educated workers for temporary employment, which residents of host countries are unwilling to take on. They often work in jobs characterized by low wages, poor occupational safety measures, job insecurity, lack of control over work processes, and exclusion from protective legislation. LMs face possibilities of various injuries, hazardous workplaces, forced labour, lack of access to health services, and a conducive environment in their workplace [3, 4].

As a result, LMs are likely to incur various diseases and injuries during employment in destination countries. These health problems have public health significance in the countries of origin; for example, male NLMs to India and their spouses were identified as one of the key risk groups of HIV in the National HIV Strategic Plan 2016–2021. Injury and mortality in young Nepalese migrant workers in Gulf Countries, Malaysia, Korea, and India have invoked public health actions, including labour rights [5].

The 72nd World Health Assembly acknowledged the health of refugees and migrants as a global priority through the acceptance of the World Health Organisation’s global action plan to promote their health [6]. Priority No. 3 of the global action plan states strengthening health monitoring and health information systems. Its main objectives are to ensure that information and disaggregated data at global, regional, and country levels are generated and that adequate, standardized, comparable records on the health of refugees and migrants are available to support policy-makers and decision-makers to develop more evidence-based policies, plans, and interventions. Health information data of LMs are limited and fragmented. There was a lack of sufficient health information about lifestyle, medical conditions, health risks, injury, and rights privilege in a survey of 1186 LMs from the Indian subcontinent in Qatar, limiting the possibility for monitoring and improving the health of LMs [7].

Nepal’s National Health Policy 2019 seeks to minimize public health risks due to migration, particularly by ensuring accessibility of health services for Nepalese migrants abroad. It directs organizational provisions for health check-ups of Nepalese migrants during pre-departure, in destination countries, and after returning to their home country. Further, the policy aims to develop a Migration Health Management Information System to keep migrant health information in all the three tiers of Government: federal, provincial, and local. It is necessary to understand better how health-related information is captured within Nepal’s health and labour migration sectors. Similarly, the government of Nepal is yet to develop the e-Health strategy based on the National Health Policy 2019 but the National e-Health Strategy 2017 and envisioned for the leveraging the use of information and communication technology in health delivery and information management [8]. In this context, the objectives of this study are to understand the management of health information of NLMs. This study will also support the Government of Nepal’s initiative to develop an migrant health information management system to improve migrant health in Nepal by elucidating status and gaps in maintaining NLMs health information.

## Methods

This is an explorative qualitative study to understand how the health data of NLMs are captured and maintained and what challenges and issues are there in recording, keeping, and sharing of health information of NLMs. The working definition of LMs in this study is: a population living in a country or area other than their country of origin, who are seeking work, are employed in the host country, or were previously seeking work or were employed but are unable to continue working and remaining in the host country [9].

Relevant governmental and non-governmental stakeholders directly or indirectly involved in assessing health or maintaining health records of NLMs were first mapped, physically visited, and any documents or information was collected in July and August 2020. Based on our preliminary mapping and information gathering of all stakeholders, we came to a consensus on the most relevant and directly involved stakeholders. The direct stakeholders are those who collect or generate migrant health data. We selected 16 stakeholders (Table 1) and key contact persons or representatives of selected stakeholders were further interviewed. We prepared a key informant interview guide to solicit information on how the health data of NLMs were maintained, what types of data are recorded, and the challenges and issues in recording and keeping such types of data. The interviews were conducted by trained study members in the Nepali language using the online (by phone) method due to the ongoing COVID-19 pandemic. Participants were informed about the purpose of research, verbal consent and permission was taken to mention the name of organization without identifying individuals. Ethical clearance was obtained from the Nepal Health Research Council ethical committee (approval number 183/2021 P) to undertake this study.

**Table 1:** Selected stakeholders for Key Informants Interview, Nepal

Stakeholders	Code of Key Informants
Department of Foreign Employment	P1
Foreign Employment Board	P2
Foreign Employment Orientation Centres	P3
Pre-Departure Medical Centre	P4
Department of Immigration	P5
Department of Health Service	P6
National Health Training Centre	P7
An insurance company	P8
Department of Passport	P9
Department of Consular Support	P10
COVID-19 Crisis Management Committee	P11
National Planning Commission	P12
National Human Rights Commission	P13
Nepali diplomatic mission in UAE	P14
Ministry of Education No Objection Certificate Unit	P15
CIWEC hospital	P16

The interviews were transcribed verbatim into the Nepali language. The transcripts were read, analyzed, and relevant information was extracted. A checklist was used to extract and disaggregate health information of NLMs into the following categories: (i) purpose of collection, (ii) sources of data, (iii) types of health indicators, (iv) time of collection, and (v) linkage of health records. Further, we manually coded the relevant information about the challenges and issues in recording and keeping health data to identify the themes.

## Results

### Status of NLMs health information management in Nepal

Table 2 shows the stakeholders who generate health records of NLMs. The FEB of the Ministry of Labour, Employment, and Social Security (MoLESS) records the deaths and disabilities of NLMs while at work abroad. The health records of deceased NLMs (destination country, cause of death, demographic information) are digitally stored using a software program. These health records are also uploaded in the FEIMS. The FEIMS is an online portal of the DoFE to collect and report migration-related data and is accessible by stakeholders including Recruitment Agencies, the Department of Passport, the Department of Immigration, the Department of Consular Services, the approved Medical Examination Centres, Pre-departure Orientation Centres, approved Insurance Companies, and approved Banks.

**Table 2:** Stakeholders in generating health records of labour migrants of Nepal

Stakeholders	Purpose	Recording procedure	Sources of data	Health indication	Collection time	Linkage to other stakeholders/ data bases
Foreign Employment Board	Provision of compensation for death and injury of LMs	Electronic (software)	LMs claims registry data	Death and injuries	After return or death	Department of Foreign Employment/FEIMS
Pre-departure Medical Centers	Medical test for eligibility of visa of the destination country	Electronic and paper	Pre-departure health assessment registry	Infectious diseases and testing of blood, faeces, urine and chest-X-ray	Pre-departure	Data sent to District Health Offices and Department of Foreign Employment
Save the Children	Screening of HIV/AIDS and TB of returnee LMs from India	Electronic	Project-based health service records	HIV and TB	After return	National Centre for AIDS and STD control
SaMI	Assessing mental health	Electronic	Project-based health service records	Mental wellbeing	After return	-
IOM	Health assessment of out-bound migrants	Electronic	Pre-departure health assessment registry	Infectious diseases and testing of blood, faeces, urine and chest-X-ray	Pre-departure	Department of Foreign Employment

The health records are derived from the documents submitted by a relative or designated beneficiary of the deceased NLM while requesting compensation for the death or injury. The cause of death of migrants is retrieved from the death certificate issued by the medico-legal institution of the destination country. The Nepali Embassy in the destination further attests to the death certificate. These documents are then used by kin of the deceased to obtain a death certificate and letter of reference from the relevant village development committee or municipality during filing for compensation at the FEB. The causes of deaths as per the FEB databases were categorized into eight groups: cardiac arrest, heart attack, traffic accident, workplace accident, suicide, murder, natural cause, and other/unidentified causes.

The Ministry of Health and Population has approved 284 medical centers to serve as pre-departure medical assessments for NLMs going abroad, mainly countries in gulf cooperation council and Malaysia. These medical centers are private and specific to international labour migrants. Health assessment of NLMs going abroad except India is a mandatory procedure. The health assessment is based on admissibility criteria set by country of destination. In general, the assessment includes medical and immunization history; detailed physical examination; laboratory and radiological investigations; and administration of vaccines.

NLMs need to pass medical tests in one of these centers after recruitment agencies conduct successful interviews. The health records of NLMs generated before pre-departure by approved pre-departure medical centers are first recorded in paper-based form and then entered into an online electronic form to be stored by the DoFE. The filled-up paper forms are sent to District Health Offices, who further report the data to the DoHS and associated governmental infectious diseases centers including the National Centre for AIDS and STD Control and National Tuberculosis Control Centers by Health Information Management System of Ministry of Health. However, District Health Offices do not maintain separate database or section for international migrants in the health management information system and the health records are not identifiable as those of international labour migrants. The data are recorded along with the data of general population.

The International Organization for Migration, Nepal Office runs migration health assessment services to assess the health status of refugees and out-bound migrants. The assessment is guided by the technical instructions provided by the intended destination countries. Two non-governmental organizations were found in assessing the health of NLMs. Save the Children conducts HIV and TB program for migrants and their spouses, focusing on NLMs who return from India in coordination with the National Centre for AIDS and STD Control. The National Centre for AIDS and STD Control (NCASC) screens the HIV/AIDS status among migrants, mainly those returning from India. Safer Migration Project (SaMi) works on the mental health of migrants and their families to cope with the difficulties of foreign employment and provides psychosocial counseling.

### Challenges in maintaining health record of NLMs

The participants have raised various issues and concerns in maintaining the health records of NLMs. These include a lack of interest to develop a unified online system; the need of competent human resources and equipment; and developing a set of health indicators for migrant health assessment.

Participants acknowledged that maintaining a systematic data information system in Nepal have been ignored while the attention has often been placed on only the financial aspect of migration. Some participants stated that some stakeholders might not benefit from the development and functioning of such a system. A unified data information system will not need many stakeholders to generate and manage migrant data.

*“We all (stakeholders) pay more attention to the financial aspect of work but not what are the health consequences and how to protect health rights of migrants; even government is not able to ensure the work and health rights of workers abroad.” P1*.

*“Some stakeholders may not want to launch such migrant health information system because their regular income can be lost.” P2*.

*“There are huge challenges to bring all stakeholders into one platform and co-ordinate because of multiple and varied interests among them.” P4*.

Since there are many stakeholders, both public and private and migrants have various health issues at different stages of migration. Participants stressed a unified electronic portal to assess migrant information, including health. Participants also pointed out that the development and running of the electronic health information system of migrants need well-trained human resources and equipment and need to be user-friendly. Some participants noted that health information could be included in the existing FEIMS, which includes migrants’ demographic and other work-related information, onto which health records can be added. Other participants said that the existing Health Management Information System (HMIS) could be expanded to include a migrant section to capture the health data of migrants.

*“Government need to develop one unified portal or software for collecting migrant data. It will make the process easy. The existing FEMIS can be further developed to include health data and linked to health information management system of Ministry of Health.” P1*.

*“We need more trained human resources. We have our system sometimes depend on one man. Sometimes old staff resist the new system because they are not trained and do not want to learn either.” P6*.

Lastly, migrants can have diverse health problems, and it is necessary to record their various health indicators, including mortality, morbidity, mental health, disability, nutritional, and behavioral factors. This will necessitate a comprehensive online tool to capture health information. Participants stated that health data are recorded before going abroad but not adequately after the return. Further, the DoFE assesses and approves the pre-departure medical centers while assessment of health status and approval of such health assessment centres should be the responsibility of the MoHP.

*“Migrants can have diverse health issues; we need to include mental health issues. Also not only health check-ups in pre-departure; health issues on arrival are more important.” P3*.

*“Migrant data need to be categorized based on types of migrant, their destination country, and purpose.” P6*.

## Discussions

In this study, we explored the health information management of NLMs and their challenges. The FEB and government-approved private assessment centers are the main stakeholders in keeping the health records of NLMs. The FEB records health data of only those migrants who got labour permit, thus excluding data of undocumented labour migrants. Government-approved private assessment centers records migrants’ health data during pre-departure health assessment. After completing the health assessment, these centers upload the data to the FEIMS. However, these health records have not been systematically kept in one database and shared among governmental health agencies due to a lack of interest in developing a unified online migrant health information system and competent human resources and equipment. The national Health Information management system under the DoHS does not effectively capture the health outcomes and determinants of NLMs despite the growing magnitude of such flows and relevance to contribution to socio-economic development.

Health records of NLMs are generated during pre-departure health assessment for eligibility of work and visa, during the processing of claims of compensation for disability and deaths of NLMs in destination countries, and during screening programs of infectious or mental diseases targeted for returnee NLMs. There is no systematic health record-keeping of NLMs going to India. Similarly, there is no systematic health screening and keeping of health records of returnee NLMs from destination countries. Further, two ministries and their departments are involved in recording migrant health data, for example, the FEB and the DoHS. This can create confusion on recording, duplication of data management, and scattering of data.

There is great potential for the health information system to effectively link with premigration health assessment medical centers where prospective migrant workers visit as a pre-requisite condition for migration to obtain health certificate for employment. The current data sharing practices from such centers are fragmented and have ineffective data sharing, record keeping and data analysis functions. The collected health record of NLMs when entered to central registries do not code for migrant status or characteristics.

There is a need for a centralized and unified information system on migration health linked with the national health information management system, but challenges towards developing such a system in a country like Nepal include technology and human resources; consensus on various health indicators and their reliable assessment; and bringing all stakeholders in one unified online portal. Further, challenges may arise since data needs to be protected for privacy and confidentiality without individual identification.

Electronic health records such as personal electronic health records are efficient and effective tools for monitoring and improving the health of migrants, especially where migrants cross the same or different borders multiple times [10]. Such tools have been developed and tested to mitigate transnational migration health issues in Mexico [11]. The lack of an effective electronic record system with the requisite data protection in Nepal inhibits understanding of health status of migrant workers. We argue that better linkages between national health information system and migrant health assessment processes at the country level are needed to shift these from being limited as an instrument of determining non-admissibility for purposes of visa issuance, to a process that may enhance public health. The importance of providing appropriate care and follow-up of migrants who fail their health assessment and the need for global efforts to enable data-collection and research are highlighted in other studies [12]. We argue that the several hundred thousand health assessments performed every year for the purposes of international labour offer an important opportunity to enhance universal health coverage.

Many high-income countries have started keeping personal electronic health records of immigrants and refugees, including relevant health indicators such as mortality, morbidity, mental health, disability, nutritional, and behavioral factors: medical history, clinical examination, vaccinations, communicable diseases, non-communicable diseases, allergies, clinical measurements, sexual health, child and obstetric care, oral health, medications, tests, follow-up, daily living activities, substance abuse, working conditions, and occupational health problems. For example, eight European countries implemented the electronic personal health record to record every migrant and refugee arriving in those countries, which have been effective in addressing migrant health issues [13]. In china, approximately 30.2% of young migrants had their health records established in inflow communities [14].

Since the FEB in Nepal records the death when death compensation is claimed for approved labour permits, the database likely excludes undocumented NLMs (those who did not obtain a labour permit from the Government of Nepal or had overstayed the contract period in the destination country). The description and categorization of causes of deaths in the database of the FEB, for example, cardiac arrest, heart attack, natural cause, and other or unidentified causes, are not scientific as per classifications of deaths by the International Classification of Diseases [15].

## Conclusions

NLMs (except going to India) obtaining labour permits are assessed for health problems before departure to check medical fitness and eligibility for visas of destination countries. However, returnee NLMs are not systematically assessed for incurred health problems upon arrival except for recording physical disability and deaths when claims are filed for compensation in the FEB of Nepal. The classification used by the FEB of causes of deaths is not scientific and does not provide the underlying causes of deaths—the present classification informs how a migrant worker died and not what caused the death. Scientific classification and proper investigation of causes of deaths and disability among NLMs are needed.

The current migrant health record keeping procedure in Nepal is fragmented and is limited as an instrument of determining non-admissibility for purposes of visa issuance. The national Health Information Management Systems does not effective capture and categorize the health record of NLMs. There is a need to effectively link national health information system with premigration health assessment centers; and potentially develop a migrant health information management system by systematically keeping health records electronically with relevant health indicators on departing and arriving NLMs at entry and exit points. Such an integrative labour migration health information system will contribute to universal health coverage and enhance the public health.

## Data Availability

Data is stored by Nepal Institute of Development Studies (NIDS). De-identified data can be made available from the first author.

## Abbreviations

DoFE: Department of Foreign Employment
DoHS: Department of Health Services
FEB: Foreign Employment Board
FEIMS: Foreign Employment Information Management System
LM: Labour migrants
MHMIS: Migration Health Management Information System
MoLESS: Ministry of Labour, Employment, and Social Security
MoHP: Ministry of Health and Population
NLM: Nepalese labour migrants
